# Effects of Vascular Photobiomodulation on Glycemic Control in Patients With Type II Diabetes Mellitus: A Prospective Single‐Arm Clinical Study

**DOI:** 10.1002/lsm.70192

**Published:** 2026-08-19

**Authors:** Leandro Augusto Almeida, Anne Missrie, Victor Augusto Ramos Fernandes, Patricia Brassolatti, Ana Laura Martins de Andrade

**Affiliations:** ^1^ Postgraduate Program in Biomedical Engineering Universidade Brasil São Paulo São Paulo Brazil; ^2^ Faculdade Israelita de Ciências da Saúde Albert Einstein São Paulo São Paulo Brazil; ^3^ Department of Research and Innovation IBRAMED Amparo São Paulo Brazil

**Keywords:** blood glucose, diabetes mellitus, low‐level light therapy, type 2

## Abstract

**Objective:**

This study aimed to evaluate the effects of laser photobiomodulation (PBM) using the systemic vascular technique (VPBM) on glycemic control in individuals with type 2 diabetes mellitus (T2DM) undergoing conventional pharmacological treatment.

**Methods:**

This prospective single‐arm clinical study was conducted at Centro Universitário UNITOP, in Palmas, Tocantins, Brazil. Individuals diagnosed with T2DM who had been receiving continuous oral hypoglycemic therapy with metformin (500 mg to 850 mg) for at least 6 months were included. PBM was applied transcutaneously over the radial artery using a low‐level laser device emitting red light, aiming to promote systemic vascular irradiation. Capillary blood glucose levels were monitored through self‐monitoring, while fasting plasma glucose and glycated hemoglobin (HbA1c) levels were analyzed in a certified clinical laboratory throughout the follow‐up period.

**Results:**

In type 2 diabetes patients, VPBM induced significant acute reductions in capillary blood glucose immediately after all sessions (from averages over 180–220 mg/dL down to 119–129 mg/dL; *p* ≤ 0.0019). It also significantly lowered baseline fasting blood glucose over the 6‐week protocol from 161.40 ± 32.63 mg/dL to 130.40 + 37.62 mg/dL (*p* = 0.0339). However, it showed no statistically significant effects on HbA1c: 7.61% to 7.65% or systolic blood pressure.

**Conclusion:**

Systemic application of low‐level laser therapy through the vascular technique, combined with standard oral hypoglycemic therapy, may contribute to improved glycemic control in individuals with T2DM. PBM associated with VPBM appears to represent a promising complementary therapeutic strategy for metabolic management in this population.

## 1 Introduction

Currently, the increased prevalence of several chronic systemic pathologies has been reported in the literature, also being associated with increased life expectancy and other factors such as physical inactivity, long working hours, inadequate lifestyle, and obesity. In this context, diabetes mellitus type 2 (DM2) stands out as a challenge to the public health system, with a significant impact on morbidity and mortality [1].

DM2 is characterized by a metabolic disorder resulting from resistance to the action of insulin and/or a relative deficiency in its secretion, resulting in chronic hyperglycemia [2]. Insulin, produced by pancreatic beta cells, plays a fundamental role in glucose regulation, especially in muscle and liver tissues [3]. In DM2, although the hormone is present, its action is impaired, a condition frequently associated with excess adiposity, especially in the abdominal region [4].

The maintenance of hyperglycemia over time is directly related to the development of microvascular and macrovascular complications, such as retinopathy, nephropathy, neuropathy, and cardiovascular diseases, which significantly compromise quality of life and increase the mortality of affected individuals [5, 6].

The management of DM2 is fundamentally based on the combination of pharmacological and non‐pharmacological interventions. Among the most commonly used drugs are metformin, sulfonylureas, and insulin [1]. At the same time, lifestyle changes such as dietary reeducation, body weight control, and regular physical activity are considered essential pillars in glycemic control. However, adherence to these measures still represents a significant challenge in clinical practice [3].

Given these limitations, interest in complementary therapies that may assist in the metabolic control of these patients has been increasing. In this scenario, PBM has been investigated as a promising strategy, with emphasis on the technique previously known as modified ILIB and currently referred to as VPBM [7]. This approach consists of the transcutaneous application of low‐level laser therapy over the radial artery region, promoting indirect blood irradiation and triggering systemic effects [4].

According to recent studies, VPBM may induce a series of relevant physiological responses, including vasodilation, improved tissue oxygenation, modulation of oxidative stress, and regulation of inflammatory processes [7]. These effects suggest a possible impact on hemodynamic parameters, especially through modulation of vascular function and microcirculation, although findings in the literature are still heterogeneous regarding the magnitude and consistency of these changes.

Regarding glycemic control, evidence indicates that photobiomodulation may promote reductions in blood glucose levels in certain contexts, possibly related to metabolic modulation and improvement of systemic conditions [8]. However, the results are still divergent, especially when medium‐term markers such as glycated hemoglobin are analyzed, which do not always show significant changes after the intervention. Consequently, a significant gap remains in the literature regarding whether the specific transcutaneous application over the radial artery (VPBM) can exert a sustained complementary effect on both acute and baseline glycemic profiles when integrated into patients' established pharmacological routines. Clinical trials evaluating its direct medium‐term impact and systemic safety within this specific therapeutic context are still scarce.

Given this scenario, the hypothesis of this study was that VPBM could contribute to improved glycemic control in patients with type 2 diabetes mellitus. The purpose of the study was to evaluate the impact of VPBM on metabolic and clinical parameters in individuals with type 2 diabetes, with the perspective of verifying its potential as a complementary therapy in the management of this chronic condition.

## 2 Materials and Methods

### 2.1 Study Design

This was a prospective single‐arm clinical study involving a total of 15 volunteers diagnosed with diabetes mellitus type 2 (DM2). The study was conducted at Centro Universitário UNITOP, located in the city of Palmas–TO.

The primary outcome was fasting blood glucose, and the secondary outcomes were glycated hemoglobin (HbA1c), capillary blood glucose, and systemic blood pressure.

### 2.2 Sample Size

The sample size calculation was performed a priori using G*Power software (version 3.1.9.4, Heinrich‐Heine‐Universität Düsseldorf, Germany), employing a one‐way repeated measures ANOVA statistical test, with interaction analysis within and between evaluation times. For the calculation, a significance level of *α* = 0.05, statistical power of 80% (*β* = 0.20), and effect size *f* = 0.5, corresponding to an effect between small and moderate, were established. Based on these parameters, a minimum sample of 15 participants was estimated.

Considering the single‐arm design, the calculation was structured for within‐subject comparisons over time, evaluating changes in fasting blood glucose between the different assessment moments.

### 2.3 Sample

Individuals of both sexes, aged 18 years or older, literate, with a clinical diagnosis of diabetes mellitus type 2, undergoing continuous metformin treatment for at least 6 months, and who agreed to sign the informed consent form (ICF) were included in the study. Participants were required to be under regular clinical follow‐up and present stability in drug treatment during the period prior to inclusion in the study.

The exclusion criteria included: 1—individuals with a history of renal failure; 2—those undergoing oncological treatment; individuals with rheumatologic and neurological diseases, such as post‐stroke, Alzheimer's disease, multiple sclerosis, and Parkinson's disease; 3—users of illicit drugs; 4—individuals diagnosed with psychiatric disorders that prevent or hinder continuation in the study; 5—individuals with heart failure, type 1 DM, and those who did not agree to sign the informed consent form. During the study period, participants were instructed to maintain their previously established medication regimen, eating habits, and physical activity pattern, and therapeutic changes without communication to the research team were not permitted.

### 2.4 Vascular Photobiomodulation Therapy

The Laserpulse device (IBRAMED) was used for the application of the VPBM technique, with a wavelength of 660 nm, continuous wave emission (CW), output optical power of 200 mW, total energy of 192 J, radiant exposure of 2742.9 J/cm^2^, and beam area of 0.07 cm^2^ (Table 1). The determination of the application parameters was based on previous studies [9]. The application time was 16 min per session. The intervention was performed twice a week for six consecutive weeks, totaling 12 sessions per participant.

**Table 1:** Photobiomodulation parameters.

Parameter	Value
Device	Laserpulse (IBRAMED)
Light source	Laser
Wavelength	660 nm
Emission mode	Continuous wave (CW)
Output power	200 mW
Beam area	0.07 cm^2^
Irradiance	2.86 W/cm^2^
Exposure time	960 s (16 min)
Radiant energy	192 J
Radiant exposure	2742.9 J/cm^2^
Number of irradiation points	1
Application technique	Contact, stationary
Irradiation site	Radial artery region

Both participants and the therapist wore protective goggles during all sessions, according to biosafety standards for the use of the technique. During the application, participants remained seated comfortably, with the left upper limb supported. To standardize the procedure, a specific bracelet was used to stabilize the laser probe, positioned over the radial artery region, maintaining a 90° angle of the equipment in relation to the skin, in order to ensure reproducibility and uniformity of the application. The probe remained immobile and in contact throughout the entire session time. In addition, the professional responsible for applying the technique underwent prior training to standardize anatomical identification and correct laser positioning over the radial artery. Adverse events were monitored before, during, and after each treatment session by direct questioning and clinical observation for symptoms including dizziness, weakness, sweating, and blurred vision.

### 2.5 Evaluation Procedures

#### 2.5.1 Data Collection

After adherence to the study through the inclusion and exclusion criteria, participants underwent an initial interview for general data collection. Information regarding age, weight, height, profession, educational level, medications in use, eating habits, and physical activity practice was initially collected. The information was recorded in individualized forms, and confidentiality of the information was guaranteed.

#### 2.5.2 Ethical and Legal Aspects

The project was approved by the Research Ethics Committee, according to Resolution No. 466/2012 of the National Health Council.

The study was conducted in accordance with the principles of the Declaration of Helsinki. Participants were informed about the minimal risks involved and were allowed to withdraw from the study at any time without prejudice to conventional treatment.

#### 2.5.3 Blood Pressure Assessment

Systemic blood pressure was measured after each session using a validated and properly calibrated sphygmomanometer by the responsible researcher. Blood pressure measurement followed the guidelines of the VII Brazilian Guideline on Hypertension (2016) [10]. For this purpose, patients were placed in a seated position, keeping their legs relaxed, uncrossed, and spine erect. A sphygmomanometer and a manual stethoscope equipped with a standard cuff were used. The measurement was performed in a standardized manner on the left arm, adjusted without looseness, approximately two to three fingers above the elbow joint. The measured region was free of clothing and rested on a surface during the procedure.

#### 2.5.4 Capillary Blood Glucose Analysis

Capillary blood glucose was used as a measure of acute glycemic monitoring throughout the intervention protocol. Measurements were performed during the first, fourth, eighth, and twelfth treatment sessions, immediately before the beginning of the VPBM application and immediately after the end of each session.

Collection was performed by fingertip puncture using portable glucometers (Accu‐Chek Active, Roche), previously calibrated before the beginning of the study. Each participant always used the same device throughout the follow‐up period in order to reduce variations between devices. The procedure strictly followed the manufacturer's recommendations regarding hygiene, disposal of lancets, and value reading. All results were recorded in an individual form by the responsible researcher immediately after each measurement.

#### 2.5.5 Glycated Hemoglobin Analysis

Glycated hemoglobin (HbA1c) was used as a marker of medium‐term glycemic control. Collections were performed at two distinct moments: 24 h before the beginning of the first intervention session (baseline assessment) and 24 h after the last session of the protocol (final assessment).

Samples were obtained through peripheral venous blood collection performed by a qualified biomedical professional in a certified laboratory. After collection, the samples were processed according to standardized laboratory protocols, and technical reports were issued to record the obtained values. All procedures followed current biosafety standards.

#### 2.5.6 Fasting Blood Glucose Analysis

Fasting blood glucose was used as the primary outcome of the study. Collections occurred at the same two moments established for HbA1c: 24 h before the beginning of the intervention and 24 h after completion of the therapeutic protocol.

Participants were previously instructed to maintain fasting for 8 to 12 h, allowing only water intake. Collection was performed through peripheral venous puncture by the responsible biomedical professional in an appropriate laboratory environment. Samples were analyzed according to standardized laboratory methods, with issuance of a technical report to record glycemic values.

### 2.6 Statistical Analysis

At the end of the collection, statistical analyses were performed by an independent researcher using GraphPad Prism 8.0 software (San Diego, CA, USA). Data normality was verified using the Shapiro–Wilk test and homogeneity of variances using Levene's test. As the variables showed normal distribution, data were expressed as mean and standard deviation. Comparisons of glycated hemoglobin and fasting blood glucose were performed using the paired *t*‐test. Capillary blood glucose, assessed before and immediately after the intervention during the 1st, 4th, 8th, and 12th treatment sessions, was analyzed using a two‐way repeated‐measures ANOVA, considering assessment timing (pre‐ and post‐intervention) and treatment session as within‐subject factors, followed by Bonferroni's multiple comparisons test. A significance level of 5% (*p* ≤ 0.05) was adopted.

## 3 Results

A total of 15 participants diagnosed with type 2 DM were evaluated, all of whom fully completed the intervention protocol, with no sample losses during follow‐up. Among the participants, 11 were female and 4 were male, with a mean age of 58.38 ± 10.79 years.

At baseline, participants presented a mean body mass index (BMI) of 28.61 ± 3.13 kg/m^2^, classified as overweight and obesity. The mean duration since diagnosis of diabetes mellitus type 2 was 13 ± 7 years, and all participants had been using metformin continuously for at least 6 months, without changes in the therapeutic regimen during the study period.

Regarding lifestyle habits, all participants reported engaging in regular physical activity, with walking being the most frequently reported exercise (87%).

Throughout the entire development of the study, few adverse effects were observed. However, it is noteworthy that two participants with phototype IV presented local burning sensation and redness at the end of the first two intervention sessions. Nevertheless, these signs were not observed in subsequent sessions, without the need to interrupt the therapeutic protocol. Systemic systolic and diastolic blood pressure were evaluated throughout the twelve consecutive sessions. Systolic blood pressure remained stable throughout the intervention period, with slight variations between sessions (Figure 1A). Observing the beginning, middle, and end of follow‐up, the mean systolic pressure values were 133.3 ± 13.6 mmHg in the first session, 125.8 ± 8.1 mmHg in the sixth session, and 127.7 ± 11.8 mmHg in the twelfth session, with no statistically significant differences observed between any of the sessions throughout the period.

**Figure 1:**
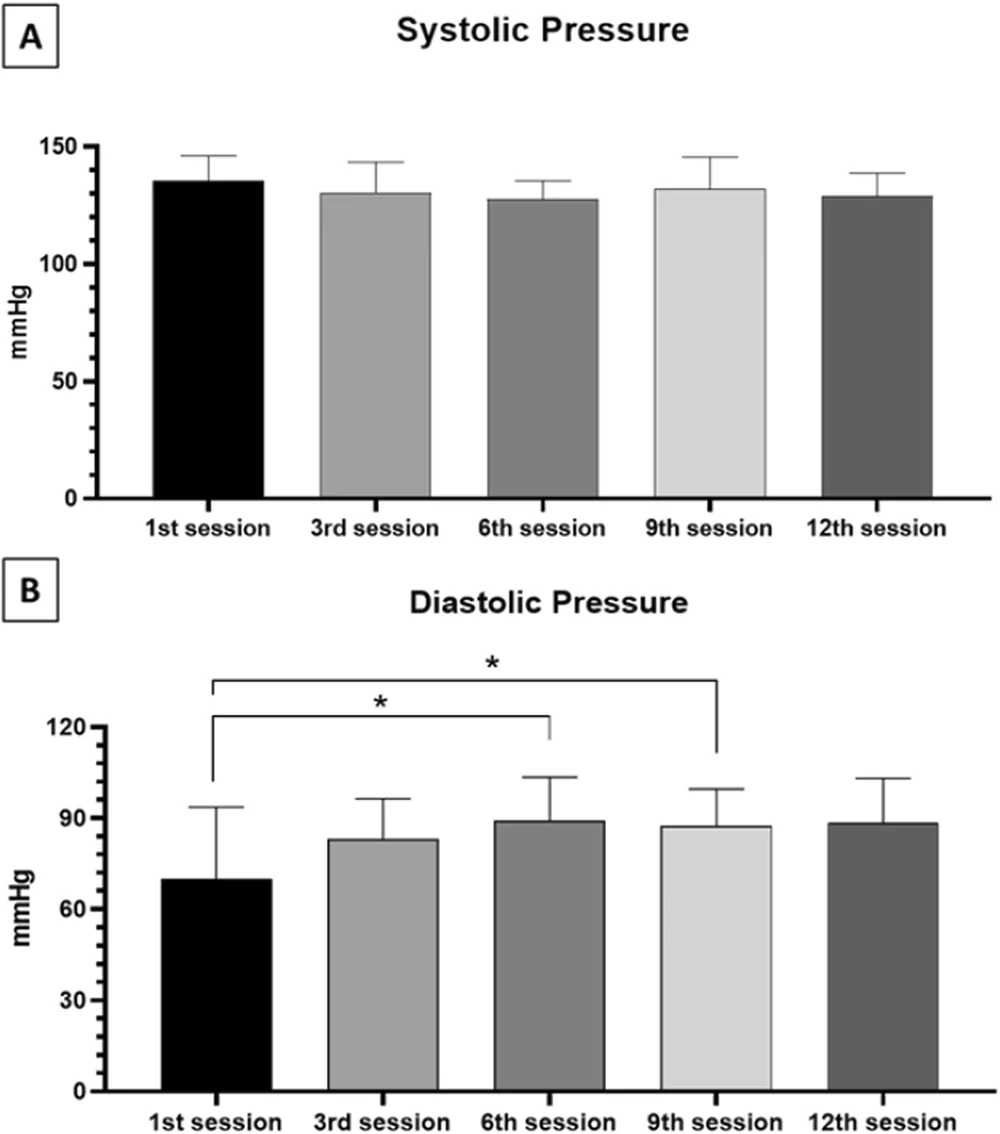
Variability of systolic (A) and diastolic (B) blood pressure throughout the intervention period.

Regarding diastolic blood pressure, an increase in mean values was observed during the intermediate sessions (Figure 1B). Diastolic pressure was 68.9 ± 24.7 mmHg in the first session, increasing to 86.9 ± 13.5 mmHg in the sixth session and 87.6 ± 11.3 mmHg in the ninth session. There was a statistically significant difference between the first and sixth sessions (*p* = 0.0079) and between the first and ninth sessions (*p* = 0.014). However, at the end of the intervention period, diastolic pressure values in the last session (87.7 ± 13.9 mmHg) did not differ significantly from those observed in the initial session (*p* = 0.059).

Complete systolic and diastolic blood pressure data, stratified by session, are presented in Table 2.

**Table 2:** Avaliação da Pressão Arterial ao Longo Das Sessões Média e Desvio Padrão.

Session	Systolic Blood Pressure (mmHg), Mean ± SD	Diastolic Blood Pressure (mmHg), Mean ± SD
1	133.3 ± 13.6	68.9 ± 24.7
3	128.8 ± 12.4	81.2 ± 13.1
6	125.8 ± 8.1	86.9 ± 13.5*
9	131.6 ± 13.7	87.6 ± 11.3*
12	127.7 ± 11.8	87.7 ± 13.9

Abbreviation: SD, standard deviation.

* *p* < 0.05 compared with Session 1.

Table 2 Blood pressure assessment across sessions: Mean and standard deviation

The analysis of capillary blood glucose demonstrated a significant reduction immediately after the ILIB intervention in all evaluated sessions.

In the 1st session, mean values decreased from 216.69 ± 67.48 mg/dL in the pre‐intervention moment to 127.46 ± 18.49 mg/dL in the immediate post‐intervention period (*p* < 0.0001). Similarly, in the 4th session, a reduction from 201.08 ± 49.96 mg/dL to 129.23 ± 19.18 mg/dL was observed (*p* = 0.0001). In the 8th session, capillary blood glucose decreased from 184.92 ± 39.89 mg/dL to 125.77 ± 24.20 mg/dL (*p* = 0.0019). In the 12th session, values changed from 220.23 ± 58.47 mg/dL in the pre‐intervention period to 119.38 ± 23.82 mg/dL in the immediate post‐intervention period (*p* < 0.0001). A significant interaction between assessment timing and treatment session indicated that the magnitude of the acute glycemic response varied across the experimental period. These findings demonstrate a significant acute effect of the intervention, with a statistically significant reduction in capillary blood glucose immediately after each application of the protocol (Figure 2).

**Figure 2:**
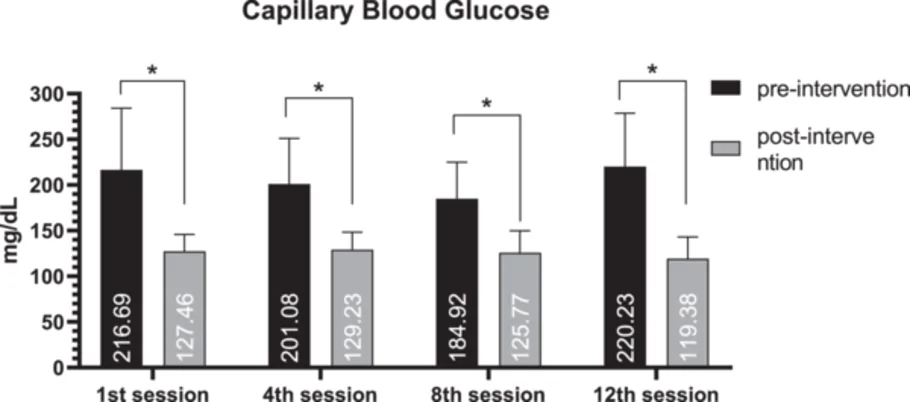
Assessment of capillary blood glucose before and immediately after the intervention in different sessions.

Fasting blood glucose was evaluated at the beginning and at the end of the intervention period, in which a reduction in mean values between the two evaluated moments was observed (Figure 3). At baseline, fasting blood glucose presented a mean of 161.40 ± 32.63 mg/dL, while at the end of follow‐up a reduction to 130.40 ± 37.62 mg/dL was observed. Statistical analysis demonstrated that fasting blood glucose at the end of the intervention was significantly lower compared to the initial value (*p* = 0.0339).

**Figure 3:**
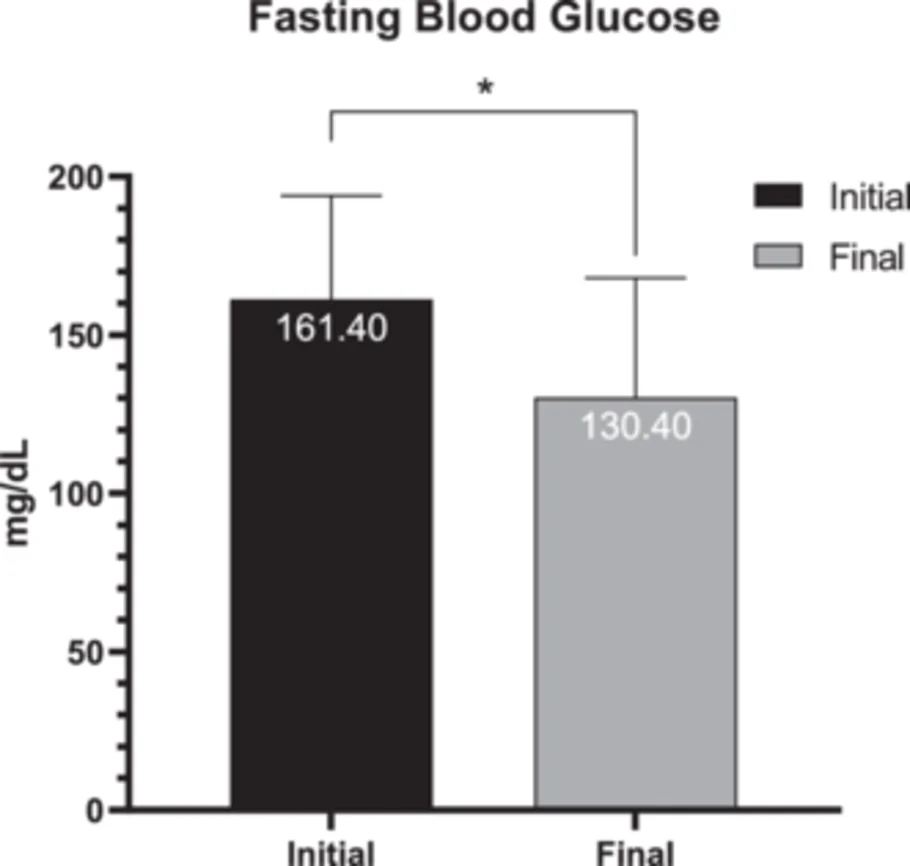
Variation in fasting blood glucose between the initial and final time of the intervention.

For glycated hemoglobin (HbA1c), the assessment was also performed at the beginning and at the end of the intervention period. At baseline, HbA1c presented a mean of 7.61%, while at the end of follow‐up the mean was 7.65%, with no expressive variation observed between the periods and no statistically significant differences (Figure 4).

**Figure 4:**
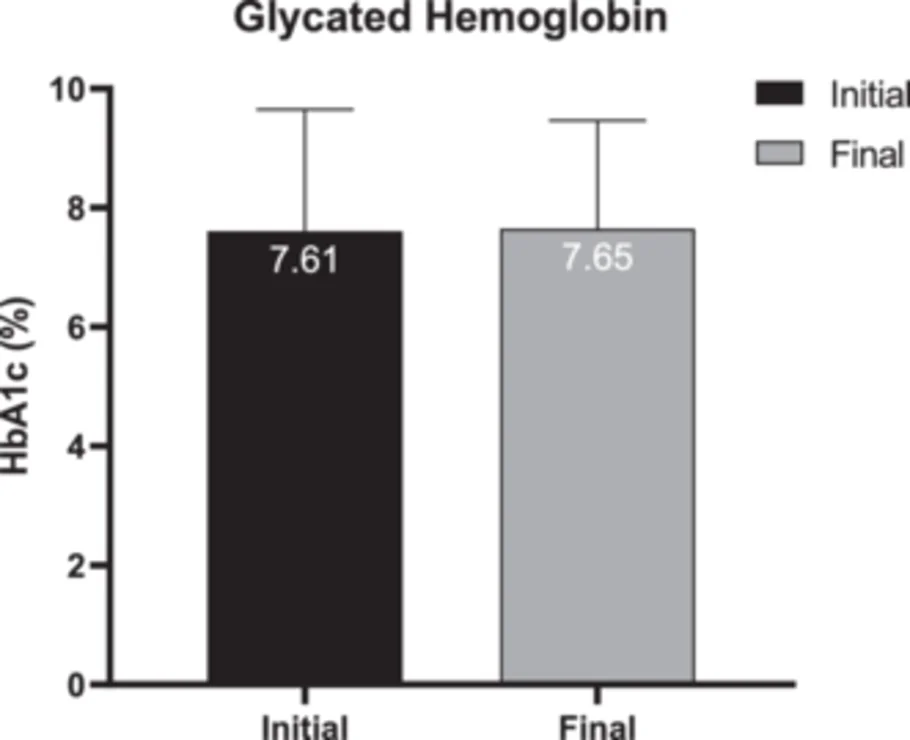
Assessment of glycated hemoglobin (HbA1c) levels at the beginning and end of the intervention.

## 4 Discussion

The present study demonstrated that the application of VPBM in individuals with DM2 using metformin was associated with lower fasting blood glucose levels and reductions in capillary blood glucose in the immediate post‐session period. However, this effect was not observed in glycated hemoglobin. These findings suggest that the intervention may induce short‐term metabolic responses, with limitations regarding the maintenance of these responses over time.

Recent studies have demonstrated that PBM stands out as a promising approach in the treatment of metabolic alterations [5]. In addition, the VPBM technique has already been discussed in literature as a complementary therapeutic resource in the management of complications associated with DM, especially regarding pain, quality of life, and functional alterations in these patients [4, 11].

Among the variables analyzed in diabetic patients, capillary blood glucose reflects immediate alterations in glucose metabolism, being sensitive to transient changes, while glycated hemoglobin represents the glycemic average of the two to 3 months prior to the assessment [12]. In the present study, a significant reduction in capillary blood glucose was observed immediately after the intervention sessions, suggesting that VPBM may be associated with acute metabolic responses. Furthermore, the reduction observed in fasting blood glucose at the end of the protocol suggests a possible association between the intervention and systemic mechanisms involved in glucose metabolism, a finding compatible with evidence describing the action of PBM in metabolic modulation and reduction of circulating glucose [5, 11]. Although the mechanisms involved are not yet fully clarified, it is believed that these effects may be related to improved cellular metabolic efficiency [9], tissue perfusion, and peripheral glucose utilization [8].

In addition, studies indicate that the technique may act on modulation of the inflammatory system, with reduction of pro‐inflammatory cytokines and increase of anti‐inflammatory cytokines, contributing to a more favorable metabolic environment [7]. Considering that type 2 diabetes is associated with a chronic inflammatory state and endothelial dysfunction, such effects may help, even in a complementary manner, in improving glucose metabolism.

However, despite the consistent acute effect observed throughout the sessions, no significant progressive reduction in baseline capillary blood glucose values was identified during follow‐up, suggesting that the effects of the intervention are predominantly transient, without sustained impact on chronic glycemic control. The absence of this cumulative effect may be related to the small sample size, the short intervention period, and the chronic and multifactorial nature of Diabetes Mellitus Type 2 itself, which involves metabolic, behavioral, and pharmacological factors directly associated with glycemic control [13].

Glycated hemoglobin, as previously discussed, is a marker that reflects medium‐term glycemic control [14]. Glycated hemoglobin, as previously discussed, is a marker that reflects average glycemic control over approximately the previous 8 to 12 weeks [14]. In the present study, no significant changes were observed in HbA1c values between baseline and the end of the intervention. This finding may be explained, at least in part, by the relatively short duration of the protocol, which lasted only 6 weeks and therefore covered a period shorter than the lifespan of erythrocytes and the time window represented by HbA1c.

Regarding hemodynamic parameters, it was observed that diastolic blood pressure presented a transient increase in some sessions, without significant differences at the end of the protocol, while systolic pressure remained stable throughout the intervention period. Although VPBM did not promote significant reductions in blood pressure levels, the findings suggest a hemodynamic safety profile, since no clinically relevant adverse impacts were observed. This aspect is particularly important, considering that Diabetes Mellitus Type 2 is associated with a higher risk of cardiovascular complications [5].

Additionally, it is important to highlight that local adverse effects in PBM are closely tied to wavelength‐related phototype tissue interactions, rather than being driven by energy dose alone. In our protocol, a 660 nm red laser was utilized; however, literature indicates that light absorption by epidermal melanin varies significantly across the electromagnetic spectrum, with shorter wavelengths facing higher absorption barriers in darker skin. Consequently, individuals with higher phototypes exhibit a higher risk of localized thermal responses—such as the redness and burning sensation observed in two of our participants—due to this wavelength‐dependent tissue interaction. This underscores that wavelength selection may be a more critical parameter than dosimetry alone when optimizing the safety profile and individualizing systemic PBM applications across diverse populations [15].

Another important factor for discussion in the treatment of type 2 DM is related to lifestyle habits, especially physical activity, which is already highlighted in the literature as an effective strategy for glycemic control when performed with adequate frequency and duration [16]. In our study, it is noteworthy that all participants performed some type of regular physical activity, a fact that also needs to be considered.

Given the above, the results of the present study should be interpreted with caution. Although acute effects on capillary blood glucose and reduction in fasting blood glucose were observed, no changes were identified in glycated hemoglobin, a marker related to chronic glycemic control. Thus, although the literature describes possible systemic effects of VPBM, such as inflammatory modulation, improvement in tissue oxygenation, and metabolic alterations [7], the findings of the present study suggest that the technique may represent a promising approach in the context of Diabetes Mellitus Type 2, especially when employed with appropriate therapeutic parameters. However, considering the complexity of glycemic control and the heterogeneity of the results available in the literature, further studies are still necessary for better understanding of its clinical and metabolic effects.

Despite the relevance of the findings, some limitations should be considered. The study included a small sample size (*n* = 15) and a single‐arm design, without a control group, sham intervention, or randomization, which limits comparative analyses and the generalization of the results. Importantly, the concomitant use of metformin and the regular practice of physical activity by all participants represent substantial confounding factors when interpreting the outcomes. Metformin primarily acts by suppressing hepatic glucose production and activating AMPK pathways, mechanisms that overlap with the metabolic adaptations triggered by structured exercise. Since physical activity independently enhances peripheral insulin sensitivity and muscular glucose uptake, the clinical improvements observed in fasting and acute capillary blood glucose cannot be exclusively attributed to VPBM, as a synergistic interaction or a dominant effect from the patients' baseline pharmacological and lifestyle regimens may have occurred. In addition, the 6‐week intervention period may not have been sufficient to promote detectable changes in markers of chronic glycemic control, such as glycated hemoglobin. Another important limitation is that dietary intake was not objectively monitored throughout the study, making it impossible to rule out the influence of nutritional factors on glycemic outcomes. Future studies with larger sample sizes, randomized comparative groups, stricter control of lifestyle‐related variables, and longer follow‐up periods are necessary to isolate the independent therapeutic efficacy of VPBM in individuals with type 2 diabetes mellitus.

## 5 Conclusion

It was concluded that the VPBM technique, applied twice a week for 6 weeks, was associated with significant reductions in capillary blood glucose immediately after each session and with lower fasting blood glucose values at the end of the follow‐up period. These findings suggest an acute metabolic effect and a possible favorable influence on baseline glycemic control in individuals with diabetes mellitus type 2.

However, no significant changes were observed in glycated hemoglobin (HbA1c) levels, a marker of medium‐term glycemic control. Thus, VPBM demonstrates potential as a complementary therapeutic strategy, especially in the acute management of blood glucose, and studies with longer follow‐up periods are necessary to evaluate sustained effects on chronic metabolic markers.

## Conflicts of Interest

The authors declare no conflicts of interest.

## Data Availability

The authors have nothing to report.
